# Changes in Refractive Errors Related to Spectacle Correction of Hyperopia

**DOI:** 10.1371/journal.pone.0110663

**Published:** 2014-11-05

**Authors:** Hee Kyung Yang, Jung Yeon Choi, Dae Hyun Kim, Jeong-Min Hwang

**Affiliations:** 1 Department of Ophthalmology, Seoul National University College of Medicine, Seoul National University Bundang Hospital, Seongnam, Korea; 2 Department of Ophthalmology, Chosun University College of Medicine, Chosun University Hospital, Gwangju, Korea; UMR8194, France

## Abstract

**Background:**

Hyperopic undercorrection is a common clinical practice. However, less is known of its effect on the change in refractive errors and emmetropization throughout the later years of childhood.

**Objectives:**

To evaluate the effect of spectacle correction on the change in refractive errors in hyperopic children less than 12 years of age with or without strabismus.

**Data Extraction:**

A retrospective cohort study was performed by a computer based search of the hospital database of patients with hyperopia, accommodative esotropia or exotropia. A total of 150 hyperopic children under 12 years of age were included. Patients were classified into four groups: 1) accommodative esotropia with full correction of hyperopia, 2) exotropia with undercorrection of hyperopia, 3) orthotropia with full correction of hyperopia, 4) orthotropia with undercorrection of hyperopia. The 4 groups were matched by initial age on examination and spherical equivalent refractive errors (SER). The main outcome measure was the change in SER (Diopter/year) in both eyes after two years of follow-up.

**Results:**

An overall negative shift in SER was noted during the follow-up period in all groups, except for the group with esotropia and full correction. The mean negative shift of hyperopia was more rapid in groups receiving undercorrection of hyperopia with or without strabismus. The amount of undercorrection of hyperopia was positively correlated to the magnitude of decrease in hyperopia in all patients (r = 0.289, *P*<0.001) and in the subgroup of patients with orthotropia (r = 0.304, *P* = 0.011). The amount of undercorrection of hyperopia was the only factor associated with a more negative shift in SER (OR, 2.414; 95% CI, 1.202–4.849; *P* = 0.013).

**Conclusions:**

The amount of undercorrection is significantly correlated to the change in hyperopic refractive errors. Full correction of hyperopia may inhibit emmetropization during early and late childhood.

## Introduction

There are two major conflicting ideas in the management of hyperopia, which puts clinicians in a certain dilemma. [1], [2] One perspective concerns the theory that eye growth and emmetropization are controlled by visual input. [3]–[5] This gives the idea that spectacle correction of hyperopia may interfere with emmetropization and leave the child with significant hyperopia. [6] The other perspective is that refractive correction of hyperopia may improve visual acuity as well as the accuracy of accommodation. [1], [2] Consequently, the recommended guidelines regarding the threshold for hyperopic correction and optimal amount of correction vary among publications [6]–[8].

Previous studies have found conflicting results of the effect of spectacle correction on emmetropization. [9]–[13] Ingram et al [10] reported that consistently wearing glasses impeded emmetropization up to the age of 42 months in normal children with hyperopia. [10] In contrast, Atkinson et al [11] demonstrated that by the age of 3 years there was no overall difference between children who were treated with partial spectacle correction and those who were not. [11] However, these studies are limited to patients with strabismus or very young infants, lacking evidence on the changes observed throughout the later years of childhood including the early school years. [9]–[12] Furthermore, these studies did not fully include various confounding factors that may affect changes in refractive errors, such as age, initial SER and strabismus [12], [13].

Thus, in this study, we aimed to determine the effect of spectacle correction on the change in hyperopia throughout early and late childhood, by comparing hyperopic children with and without strabismus matched by age and refractive errors, who received different amounts of spectacle correction.

## Methods

A retrospective search was performed of patients under the age of 12 with the diagnosis of hyperopia with or without accommodative esotropia or exotropia who first visited Seoul National University Bundang Hospital between November, 2003 and October, 2013. Patients were included if their full cycloplegic refractive errors in both meridians with the greatest and least amounts of hyperopia were +1.50 diopters (D) or more in both eyes and had a follow-up period of more than 2 years. Patients were excluded if they previously had a history of other ocular pathologies, surgeries, systemic or neurologic abnormalities or a follow-up period of less than 2 years. Full hyperopic correction was defined as the full correction of cycloplegic refractive errors or a reduction of hyperopia ≤0.75 D of their cycloplegic refractive errors in both eyes. Undercorrection of hyperopia was defined as an undercorrection of ≥1.00 D of their cycloplegic refractive errors. Patient records/information was anonymized and de-identified prior to analysis. Approval to conduct this study was obtained from the Institutional Review Board of Seoul National University Bundang Hospital, and adheres to the Declaration of Helsinki.

### Group classification

Patients were allocated into four groups as follows.

(Group 1) Accommodative esotropia with full hyperopic correction.
(Group 2) Exotropia with undercorrection of hyperopia.
(Group 3) Orthotropia with full hyperopic correction.
(Group 4) Orthotropia with undercorrection of hyperopia.

Records were acquired from 1,893 patients with a registered diagnosis of hyperopia, 399 patients with accommodative esotropia and 4,212 patients with exotropia in our electronic medical record database. Exotropia is far more common than esotropia in Asians, and as we are a tertiary referral hospital renowned for strabismus surgery, a large majority of patients are referred for exotropia. [14] All patients in each group were subdivided according to their initial age at examination and SER of the more hyperopic eye in a positive number. Then patients in each group were randomly selected from each subgroup to be matched for their initial age at examination and SER of the more hyperopic eye within ±0.75 D. A substantial number of patients were excluded during the double matching of both age and SER due to discrepancies of the mean age and SER among groups. Finally, a total of 150 patients were included: 40 patients in the esotropia group with full correction of hyperopia, 40 patients in the exotropia group with undercorrection of hyperopia, 35 patients in the orthotropic group with full correction of hyperopia and 35 patients in the orthotropic group with undercorrection of hyperopia.

### Patients’ characteristics

Patients’ characteristics were acquired at their initial visit and follow-up examinations, including age, gender, best corrected visual acuity (BCVA), cycloplegic refractive errors and ocular deviation. Cycloplegic refraction was performed in all patients, 45 minutes after 3 to 5 instillations of one drop of cyclopentolate 1% and was reported as SER values. Spectacles were prescribed on the basis of cycloplegic refraction, if necessary, and full correction of the astigmatic refractive error was prescribed. The amount of undercorrection was defined as the difference between spectacle prescription minus cycloplegic refractive errors in SER values and symmetrical reduction was performed on both eyes. Spectacle prescriptions were variable on an individual basis, considering various factors of concomitant strabismus, initial age, BCVA, astigmatism, amblyopia and clinician preferences. Accommodative esotropes were prescribed full cycloplegic correction at their initial examinations. During follow-up, the maximum permissible reduction maintaining stable alignment was a reduction of up to 0.75 D in both eyes. Undercorrection of hyperopia of 1.00 D or more than the full cycloplegic hyperopic refraction was typically recommended to achieve the best BCVA and ocular alignment in exotropes. Orthotropic patients with anisometropic amblyopia who underwent occlusion therapy were prescribed spectacles with an undercorrection of 1.50 D or less. If spectacles were not prescribed, the amount of undercorrection, or hyperopic reduction, was documented as the SER of cycloplegic refraction with a negative value. Anisometropia was defined as a spherical or cylindrical difference of more than 1.50 D. Amblyopia was defined as a difference of 2 lines or more between monocular BCVA. Part-time or full-time occlusion was implemented to treat amblyopia according to the severity of amblyopia and was tapered with maintenance occlusion therapy for several months when amblyopia was no longer evident in follow-up examinations.

### Main outcome measures

Main outcome measures were changes in SER after 2 years of follow up. The change in SER (D/year) was compared between groups. Factors associated with changes in SER, including initial age at examination, initial SER, type of strabismus, amount of undercorrection, initial age and duration of spectacle wear, astigmatism, anisometropia and amblyopia were evaluated.

### Statistical analysis

Statistical analyses were performed using SPSS for Windows (Ver. 18.0, Statistical Package for the Social Sciences, SPSS Inc., Chicago, IL). SER data were compared between groups with one-way analysis of variance and multiple comparisons were corrected using the Bonferroni method. The influence of various factors on the magnitude of the absolute change in SER was examined with logistic regression models and Pearson’s correlation coefficients. The change in SER was assessed using a 2-level categorical variable indicating either a more negative shift than −0.30 D/year or ≥−0.30 D/year. *P* values of <0.05 were considered statistically significant.

## Results

The patients’ characteristics are summarized in Table 1. There were no significant differences in the age of patients at the time of initial examination, initial SER of both eyes, the duration of spectacle wear, or the frequency of anisometropia and amblyopia among the four groups. Astigmatism was significantly larger in the group with orthotropia compared to the group with esotropia or exotropia (*P*<0.05 by one-way ANOVA, post-hoc Bonferroni).

**Table 1:** Patients’ characteristics and change in refractive errors of age-matched and spherical equivalent refractive error-matched groups of hyperopic patients who received full or undercorrection of hyperopia, with and without strabismus.

		Strabismus		Orthotropia		*P* value
		Esotropia	Exotropia			
		Fullcorrection(n = 40)	Undercorrection(n = 40)	Fullcorrection(n = 35)	Undercorrection(n = 35)	
Age (y)		5.2±2.8(1.1∼12.1)	5.3±2.9(1.1∼12.0)	5.1±2.0(1.3∼11.3)	5.6±2.2(2.1∼12.3)	0.829^a^
Male		16 (40%)	16 (40%)	19 (54%)	18 (51%)	0.183^b^
Initial SER (D)	*More* *hyperopic* *eye*	3.54±1.61(1.50∼7.25)	2.98±1.65(1.50∼7.50)	3.71±1.46(1.50∼6.75)	3.64±1.81(1.38∼7.50)	0.195^a^
	*Less* *hyperopic* *eye*	3.01±1.46(1.50∼6.50)	2.38±1.33(1.50∼6.50)	3.19±1.24(1.50∼5.50)	3.14±1.70(1.50∼6.75)	0.056^a^
Astigmatism		1.01±0.95(0.00∼3.50)	1.27±1.13(0.00∼3.50)	2.16±1.06(0.00∼4.00)	1.76±1.02(0.00∼3.50)	0.017^a^
Anisometropia		2 (5%)	6 (15%)	1 (3%)	2 (6%)	0.621^b^
Amblyopia		6 (15%)	2 (5%)	4 (11%)	2 (6%)	0.308^b^
Undercorrection (D)		–0.03±0.14	–1.89±0.89	–0.26±0.31	–1.63±0.61	<0.001^a^
		(–0.75∼0.0)	(–5.00∼–1.00)	(–0.75∼0.0)	(–3.00∼–1.00)	
Spectacle wear (n)		40 (100%)	33 (82.5%)	35 (100%)	32 (91%)	0.626^b^
Changein SER (D/y)	*More* *hyperopic* *eye*	0.03±0.53(–0.89∼1.59)	–0.43±0.46(–1.38∼0.62)	–0.15±0.43(–1.19∼0.66)	–0.48±0.48(–2.33∼0.13)	<0.001^a^
	*Less* *hyperopic* *eye*	0.14±0.71(–2.05∼2.30)	–0.27±0.37(–0.96∼0.74)	–0.02±0.43(–0.98∼0.90)	–0.36±0.38(–1.29∼0.60)	<0.001^a^

Mean ± standard deviation (range); y = years; D = diopters; SER = spherical equivalent refractive error.

a One-way ANOVA;

b Linear-by-linear association.

### Changes in refractive errors

An overall negative shift in SER of both eyes was noted during the follow-up period in all groups, except for the group with esotropia and full correction (Table 1). The mean negative shift of hyperopia in the more hyperopic eye (D/year) was more rapid in groups receiving undercorrection of hyperopia with (–0.43±0.46, group 2) or without strabismus (–0.48±0.48, group 4), compared to those with full corrected hyperopia with (0.03±0.53, group 1) or without strabismus (–0.15±0.43, group 3) (*P*<0.05 by One-way ANOVA, post-hoc Bonferroni). The mean negative shift of hyperopia in the less hyperopic eye (D/year) was more rapid in groups receiving undercorrection of hyperopia with (–0.27±0.37, group 2) or without strabismus (–0.36±0.38, group 4), compared to those with full corrected hyperopia with (0.14±0.71, group 1) or without strabismus (–0.02±0.43, group 3) (*P*<0.05 by One-way ANOVA, post-hoc Bonferroni) (Table 1).

In the subgroup of patients with orthotropia, the difference of the mean change in SER between group 3 and 4 was 0.33D/y (P = 0.029, effect size 0.7) in the more hyperopic eye and 0.34D/y in the less hyperopic eye (P = 0.029, effect size 0.8).

### Factors associated with changes in spherical equivalent refractive error (SER)

**Figure 1:**
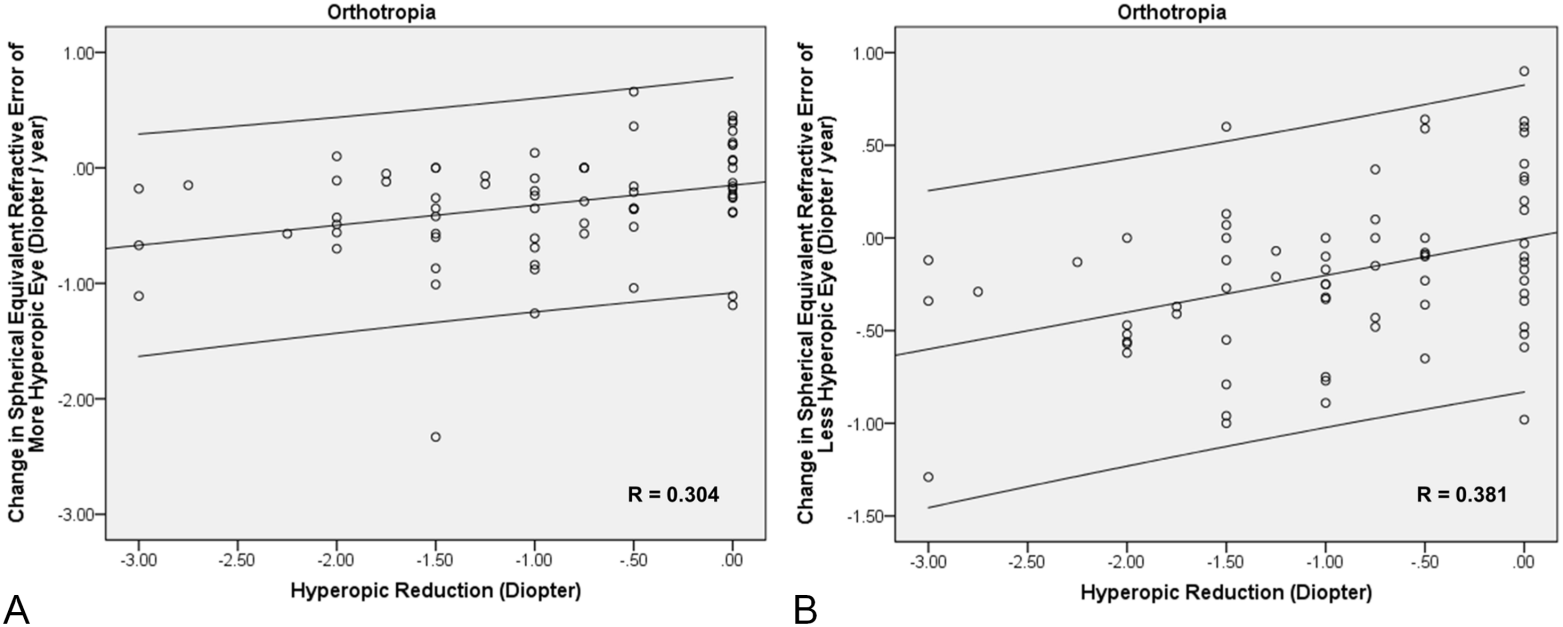
In the subgroup of orthotropic patients with hyperopia who received full or undercorrection of hyperopia, there was a positive correlation between the amount of undercorrection and the magnitude of change in hyperopia (A) in the more hyperopic eye (r = 0.304, R^2^ = 0.092, *P = *0.011, y = −0.15+0.17*x) and (B) in the less hyperopic eye (r = 0.381, R^2^ = 0.145, *P* = 0.001, y = −2.96E–3+0.2*x).

There was an overall positive correlation between the amount of undercorrection and the magnitude of change in SER of the more hyperopic eye (r = 0.289, *P<*0.001) and less hyperopic eye (r = 0.344, *P<*0.001) in all patients. Within the subgroup of orthotropic patients who received full or undercorrection of hyperopia, there was a positive correlation between the amount of undercorrection and the magnitude of change in SER of the more hyperopic eye (r = 0.304, *P = *0.011) and less hyperopic eye (r = 0.381, *P* = 0.001, effect size 0.8) (Figure 1). Within the subgroups of patients with esotropia or exotropia, there was no significant correlation between the amount of undercorrection and the magnitude of change in SER in both eyes (*P*>0.05).

The amount of initial hyperopia, astigmatism, anisometropia and age at initial examination was not significantly related to the magnitude of change in SER of both eyes in overall patients, as well as in subgroups.

Multiple logistic regression models including initial age at examination, gender, initial SER, amount of undercorrection, astigmatism, anisometropia and presence of amblyopia within the subgroup of orthotropic patients who received full or undercorrection of hyperopia revealed that the amount of undercorrection was the only factor associated with a more negative shift in SER of the more hyperopic eye (odds ratio 2.414, 95% confidence interval 1.202–4.849, *P* = 0.013) and the less hyperopic eye (odds ratio 2.467, 95% confidence interval 1.174–5.183, *P* = 0.017).

## Discussion

The major strength of our study is that factors mainly known to affect changes in refractive error such as age, initial SER, and strabismus were matched among groups. [12], [13] In addition, children of various ages up to 12 years were included, which allowed us to investigate hyperopic refractive errors in children throughout the late years of emmetropization. The results of our study suggest that spectacle correction may impair the normal developmental regulation of hyperopia throughout early and late childhood. In our study, the amount of undercorrection of hyperopia was the only factor related to the magnitude of the negative shift in hyperopia.

Previous studies have sought to identify an association between wearing spectacles and the change in hyperopia in patients with and without strabismus. [10]–[13] In hyperopes without strabismus, Ingram et al [10] reported that consistently wearing glasses (2.00 D less than their cycloplegic refraction) impeded emmetropization in both eyes of normal children up to the age of 42 months. [10] In contrast, Atkinson et al [11] demonstrated that the negative shift in hyperopia as experienced by the age of 3 years showed no overall difference between children who were treated with partial spectacle correction (1.00 D less than their cycloplegic refraction) and those who were not. [11] Aside from the controversy on these conflicting results, these studies included only young children from 1 to 4 years of age. However, in clinical practice, hyperopia is not treated or even noticed unless it is accompanied by strabismus, deficient accommodation, or decreased visual acuity. [6] Notably, decreased visual acuity is difficult to identify in preverbal children. In addition, although the majority of emmetropization takes place in the first year after birth, [1], [5] progressive changes in refractive errors have been documented until late childhood and adolescence. [12], [13] Thus, the results of our study support the association between spectacle wear and the change in hyperopic refractive errors among children throughout the later years of emmetropization up to 12 years of age, regardless of the presence of strabismus.

In contrast to the scarce literature concerning hyperopic children without strabismus, [13] there are a considerable number of reports on refractive error changes in children with strabismus. Longitudinal studies of accommodative esotropia have revealed that refractive errors show a slow negative shift over time and these changes were mostly related to the age when spectacles were first prescribed or the initial degree of hyperopia. [12], [13] Others found that spectacle wear itself did not affect refractive changes in accommodative esotropia. [10], [15] As for exotropia, a significant myopic shift over time compared to similarly aged non-strabismic children were reported in a population-based study. [16] However, neither surgical correction nor an overcorrecting minus lens altered the refractive outcome in exotropes. [17], [18] Among children treated for amblyopia, the more negative shift toward emmetropia was associated with better ocular alignment. [19] In our study, the mean negative shift of SER in the more hyperopic eye was similar in undercorrected exotropes (–0.43 D/year) and orthotropes (–0.48 D/year), which was significantly more negative than the changes in fully corrected subjects. Interestingly, the mean negative shift of hyperopia in fully corrected orthotropes (–0.15 D/year) was intermediate between that observed in fully corrected esotropes and undercorrected exotropes/orthotropes. This implies that full correction of hyperopia and esotropia may independently impede axial growth of the eye and the reduction of refractive errors [10], [20].

Astigmatism was significantly less in patients with strabismus compared to patients with orthotropia. This is probably because of the need for hyperopic correction in orthotropic subjects with significant astigmatism. However, in our study, linear regression models revealed no significant effect of astigmatism on refractive error changes. The overall change in astigmatism during the follow-up period did not differ among the 4 groups.

Some limitations of our results need to be considered. First, this was a retrospective study with a relatively few patients in the orthotropic group. These patients usually do not need glasses and are seldom referred to a tertiary referral hospital. Furthermore, most of these patients receive only partial hyperopic corrections. We presume that this is in accordance with the general clinical practice in ophthalmology clinics. [6] Therefore, it would be difficult to include a larger patient population unless a randomized controlled trial is performed. Second, the parental history of refractive errors was not included. The heritability of SER ranges from 75% to 88%. [21] To overcome these factors, we matched the initial SER at a certain age in all groups. Third, all patients with esotropia were fully corrected while all patients with exotropia were only partially corrected, owing to the classical guidelines in our clinical practice. To maintain ocular alignment, accommodative esotropia must be fully corrected, while exotropia benefits from undercorrection. [6], [22] Thus, the amount of undercorrection was closely associated with the presence of strabismus. Because of this strong correlation among factors, multiple regression models could not be used to analyze the amount of undercorrection in patients with strabismus. Finally, accommodative function was not regarded, which is an important component to be considered in prescribing for hyperopia. BCVA may depend on various factors such as age, the magnitude of hyperopia, phoria, accommodative convergence/accommodation ratios and accommodative functions. [2] These factors may predict whether a particular refractive correction will benefit a hyperopic child. There is a possibility that orthotropic patients in this study who tolerated full correction of hyperopia had low accommodative amplitudes, although they did not complain of any significant visual symptoms. Further studies on accommodative amplitudes and facilities among hyperopic children as related to spectacle correction may clarify this issue.

In conclusion, full correction of hyperopia may inhibit the negative shift of hyperopia over time and normal emmetropization during childhood, regardless of the presence of strabismus. The amount of undercorrection observed among the study population was significantly correlated with the change in hyperopic refractive errors during early and late childhood.
